# Short-term ketamine use in bipolar depression: a review of the evidence for short-term treatment management

**DOI:** 10.3389/fpsyt.2023.1322752

**Published:** 2023-12-08

**Authors:** Alina Wilkowska, Wiesław Jerzy Cubała

**Affiliations:** Department of Psychiatry, Faculty of Medicine, Medical University of Gdańsk, Gdańsk, Poland

**Keywords:** bipolar depression, ketamine, short-term treatment, esketamine, arketamine

## Abstract

Bipolar depression constitutes a major problem in psychiatry. It correlates with high suicidality, treatment resistance, chronicity, and poor quality of life. Registered treatment for bipolar depression is limited and insufficient. There is an urgent need for implementing new therapeutic strategies. Intranasal ketamine’s enantiomer—esketamine is a novel rapid-acting antidepressant with proven efficacy in treatment-resistant depression. Research on bipolar depression, although not as comprehensive, indicates that it may be a viable and safe substitute with minimal risk for mood polarity changes. Reports suggest that ketamine treatment in bipolar depression may reduce suicidal tendencies, decrease anhedonia, and alleviate anxiety. Ketamine’s mood-stabilizing properties are also hypothesized. In this narrative review, we focus on ketamine use as an add-on to standard medication for the acute treatment of bipolar depression.

## 1 Introduction

Bipolar depression is a chronic and debilitating disease with a high risk of suicide attempts and completed suicide (1, 2). Approximately one-third of patients do not respond to two treatment options and are considered treatment resistant (3, 4). The time being ill in bipolar disorder is mostly consumed by depressive episodes which makes it particularly burdensome for patients and their families (2). There are currently five Food and Drug Administration (FDA)—approved treatment options for bipolar depression: olanzapine plus fluoxetine, quetiapine, lurasidone, cariprazine, and lumateperone (5). The European Medicines Agency (EMA) has authorized only quetiapine for the treatment of major depressive episodes in bipolar disorder (6).

Ketamine, an N-methyl-D-aspartate (NMDA) receptor antagonist, a novel rapid-acting antidepressant has been studied during the last decade mostly in major depressive disorder (MDD) (7). Intranasal esketamine is approved by the FDA for two indications. One is an add-on treatment of treatment resistant major depressive disorder and the second is an acute short-term treatment for the rapid reduction of depressive symptoms in moderate to severe depressive episodes constituting psychiatric emergency also in combination with an oral antidepressant (8). The second indication was based on the results of ASPIRE I and ASPIRE II studies (9, 10).

Initial research did not demonstrate the effectiveness of the treatment in bipolar depression (11). Still, according to a very recent systematic review (12) eight included studies suggest that ketamine is a safe and effective add-on treatment for bipolar disorder with an overall pooled response rate of 48%. The reported antisuicidal effect of ketamine is explicitly significant for bipolar depression where suicide risk is particularly high (13). The analysis of the antisuicidal effect of ketamine in studies included in the mentioned systematic review revealed a large reduction in suicidal symptoms in all of them (11). Four of the eight included studies investigated single ketamine administration, three used multiple dosing and one investigated esketamine (14).

This is a narrative review of recent literature focusing on ketamine and esketamine management in bipolar depression. We have included papers available up to October 13th 2023 using PubMed and Web of Science. The search terms were “bipolar depression,” “suicidality and bipolar depression “emergency psychiatry,” “ketamine,” “esketamine,” and “arketamine.” Human studies were selected based on their high methodological quality, and on how informative and innovative they were. Both original research papers and reviews are included. Randomized controlled trials (RCTs) and prospective research were prioritized, but cautiously selected open-label reports and registries were also included. A few case reports were mentioned when other trials were not available.

First racemic ketamine studies in bipolar depression with single and multiple dosing are presented. Then the studies investigating the efficacy and safety of ketamine’s enantiomers—esketamine and arketamine in bipolar depression are discussed. Finally, the molecular mechanisms of ketamine in bipolar depression and suggestions for future approval in bipolar disorder are stated.

## 2 Studies investigating ketamine in bipolar depression

### 2.1 Racemic ketamine

#### 2.1.1 Intravenous

##### 2.1.1.1 Single infusion

Unlike esketamine, racemic ketamine which is a mixture of two enantiomers esketamine and arketamine is not officially approved in psychiatry. However, it is used in many countries, and few ketamine registries are continuing (NCT0339660, NCT0533907, NCT05209217). It constitutes an interesting alternative for patients with limited access to esketamine NS (nasal spray), which is much more expensive. The first ketamine studies in bipolar depression took place over a decade ago and investigated a single intravenous infusion dosed 0,5 mg/kg together with standard-of-care mood-stabilizing treatment. Two of them were randomized placebo-controlled (15, 16), one was a parallel vs. midazolam (17) and two were open-label trials (15–19). All five studies except one (17) included treatment resistant patients. The response rate in those trials varied from 24.5% which increased to 51% after 7 days (19) to 79% (16). The efficacy of a single infusion did not extend over 2 weeks. The treatment was safe, and quite well tolerated no serious adverse events were observed. In one of the 5 studies, one patient in the treatment group and one in the placebo group presented a switch to manic symptoms (15). A small open-label trial focused on the cognitive and antidepressant effects of a single ketamine infusion in 18 subjects with bipolar depression (18). An exploratory analysis of data from the discussed studies by Diazgranados et al. (15) and Zarate et al. (16) aimed to investigate the anti-fatigue effects of ketamine in patients with bipolar depression and found a maximum response rate of 65% during the trial. The largest difference in anti-fatigue effects between placebo and ketamine was observed on day 2. Interestingly, the effect remained significant after controlling for changes in non-fatigue depressive symptoms (20).

##### 2.1.1.2 Multiple infusions

The following trials investigated multiple infusions of ketamine in treatment resistant bipolar depression. According to the most recent systematic review (12) three studies investigated multiple ketamine infusions in patients with treatment-resistant bipolar depression (TRBD). All of them were open-label observational trials. The first one used 6 ketamine infusions over 12 days to treat 19 patients (21). The second one was a study from our group with 8 infusions over 4 weeks in 13 patients (22). In the most recent trial, 66 patients started with two ketamine infusions and in case of inadequate response, they had an additional two with the increased dose of 0.75 mg/kg. The authors also found a significant decrease in suicidality and anxiety symptoms (23). The response rate in those three studies varied and reached between 35 and 73%. The treatment was generally well tolerated, 4 cases of the hypomanic switch were reported (23). A former open-label study by Zhuo et al. (24) with nine ketamine infusions over 3 weeks in 38 patients with bipolar depression showed an antidepressant effect after the first three doses, with subsequent worsening of depressive symptoms and full relapse by the third week. The reason for this discrepancy could be the small sample sizes or higher level of resistance in the study group, all patients together with mood stabilizers received antidepressants which could also affect the results (24). In phase 1 of the open-label study by Chen et al. Nineteen patients with bipolar depression and 30 with MDD were enrolled. They received two ketamine infusions on day 1 and day 4 of the study. The response rate was slightly higher in the bipolar group and reached 68%, whereas in the MDD group, it was 63% at day 7 compared to baseline (25).

#### 2.1.2 Oral/sublingual racemic ketamine

According to the most recent systematic review (26), data on ketamine administered orally in bipolar depression is very limited. There are only two studies mentioned. The first one included 26 patients with MDD or bipolar depression who were administered 100 mg/mL sublingual ketamine every 2–3 days or weekly for up to 6 months together with antidepressant or mood stabilizing medications. Twenty patients achieved improvement, but the bipolar group was not analyzed separately. We cite this study because it is one of the two published reports on oral ketamine in BD, although it is not a short-term treatment trial (27). The other one is a case report describing two patients with bipolar depression who improved significantly after oral ketamine up to 3 mg/kg with amitriptyline 200 mg and quetiapine 100 mg, the treatment was well tolerated. The length of ketamine treatment is not stated (28).

### 2.2 Esketamine

#### 2.2.1 Nasal spray

The results of two phase 3 randomized clinical multicenter trials on the effect of intranasal esketamine in MDD - ASPIRE I and ASPIRE II (9, 10) led to FDA esketamine NS (Spravato) approval for moderate to severe MDD episodes, as an acute short-term treatment, for the rapid reduction of depressive symptoms which according to clinical judgment constitute a psychiatric emergency. Esketamine NS should be co-administered with oral antidepressant therapy and the recommended dose is 84 mg twice a week for 4 weeks (8). EMA esketamine ESK-NS summary of product characteristics does not contraindicate ESK-NS use among subjects with bipolar disorder, but it suggests a careful evaluation between the risk and benefits of its application and watchful safety assessment in this condition (29).

The evidence on the use of ESK-NS in bipolar depression is very limited. We managed to identify 3 studies. The first one is a recent open-label double-arm observational trial investigating the effect of two doses (28-84 mg) of intranasal esketamine per week during the first 4 weeks and one dose a week in the following 2 months. Thirty-five patients with treatment resistant depression (TRD) and 35 with TRBD were included. The most common definition of treatment resistance is the failure to achieve a satisfactory response to two trials of antidepressant or mood-stabilizing medications administered in adequate doses and for a sufficient period.

The authors found a significant improvement in depressive symptoms at 1 month and 3 months compared to baseline. The response rates were very similar in both groups. Interestingly, esketamine caused a more pronounced anxiolytic effect in patients with bipolar depression. There was only one case of an affective switch observed in the bipolar group and one case of psychomotor agitation, other serious adverse events were not observed (14). The other two reports concern case descriptions with one-year observation, thereby not encapsulating short-term treatment effects. However, their inclusion has been deemed pertinent considering the paucity of data. The first one is a case report of a patient with bipolar depression and multiple comorbidities. The patient initially received 56 mg for the first 2 doses and continued with 86 mg twice weekly for 4 weeks and subsequently once a week for 1 year. Throughout the study, lurasidone and bupropion were coadministered. The improvement was moderate, and no manic switch was observed (30). The second case report describes a patient with severe bipolar depression, borderline personality disorder, and a history of substance abuse disorder, monitored over a period of 1 year. The patient started with 56 mg twice a week for 4 weeks and continued with 56 mg once a week. Lithium, lurasidone, quetiapine, and fluoxetine were coadministered. Clinical response was observed after 3 months, and remission occurred at 12 months. Reduction in suicidality, anxiety, and binge eating symptoms was also observed. No affective switch or substance use disorder symptoms were observed during the study (31).

#### 2.2.2 Subcutaneous

A retrospective real-world study investigated subcutaneous esketamine treatment in 70 patients with suicidality due to depressive episodes in MDD (*n* = 39) or BD (*n* = 31). Esketamine doses were 0.5 mg/kg at the start and then increased to 0.75 mg/kg and 1.0 mg/kg according to the therapeutic response. Standard of care oral treatment was continued. The patients received ketamine injections once a week for 6 weeks. A significant and rapid anti-suicidality effect was observed in both MDD and BP groups. The observed tolerability profile was favorable. Authors report that adverse events were mild, and no patients withdrew from treatment due to poor tolerability. Transient blood pressure increase occurred in about 30% of patients (32).

### 2.3 Arketamine

#### 2.3.1 Intravenous

We identified one pilot study with the use of arketamine as an adjunct treatment in 6 patients with bipolar I and II disorder. The patients received 2 intravenous (IV) infusions 1 week apart. The first infusion dose was 0.5 mg/kg, the second was increased to 1 mg/kg. The authors observed a reduction in the total Montgomery–Åsberg Depression Rating Scale (MADRS) score and item 10 MADRS score describing suicidality. The response rates were lower than those seen in racemic ketamine trials and reached 33.3% at 24 h and 16.6% at 7 days. They did not report any manic or dissociative symptoms (33).

## 3 Antisuicidal effect of ketamine in bipolar depression

The antisuicidal effect of racemic ketamine was investigated in a mentioned single infusion RCT study by Grunebaum et al. (17). The authors did not observe a statistically significant anti-suicidal effect of IV ketamine compared to the control which was IV midazolam. In fact, in two cases suicidality increased and one patient had a suicide attempt in the second month after the infusion. No other studies used midazolam as a control, so it is difficult to compare the results (34). Another RCT investigated suicidal symptoms in 156 patients including 52 with BD and found a statistically significant decrease in the group receiving two racemic ketamine infusions compared to placebo by day three. The response rate in the BP group was 84% vs. 28% in the placebo group, moreover, in the MDD group response rate was 42% vs. 37% in the placebo group (35). The previously referenced open-label study from Canada demonstrated a marked amelioration in suicidal symptoms after four ketamine infusions (23). In the above-described retrospective study by Surjan subcutaneous esketamine had a rapid anti-suicidal effect in patients with BD and MDD (31). Additional research has examined the anti-suicidal effects of ketamine in individuals with MDD and those diagnosed with other conditions, wherein ketamine administration was characterized as an intervention in emergency department settings (36, 37). According to an international expert opinion, there is evidence for the antisuicidal effect of ketamine and esketamine in TRD in single and repeated doses (38). ASPIRE I and II MDD studies although did not show the difference in terms of diminishing suicidal ideation, they still provided the basis for FDA approval of esketamine for the treatment of major depressive disorder with suicidal ideation or behavior (9, 10, 38).

## 4 Ketamine safety and tolerability in bipolar depression

Studies investigating ketamine and its enantiomers use in bipolar depression suggest it is mostly safe and well-tolerated. Reported side effects (i.e., feeling dizzy, cognitive impairment, dissociation, nausea, headache, odd sensations, flatulence, and blurred vision) were transient. The cases of discontinuation were rare (14, 16, 18, 19, 21, 23, 24, 32, 35). One report of increased suicidality was described above and seems an isolated event (17). Another case report described non-suicidal self-harming behaviors with increased impulsivity in a patient with bipolar depression and comorbid borderline personality disorder treated with an add-on IV ketamine (39). The incidence of a manic or hypomanic switch attributable to ketamine therapy appears to be minimal. Conversely, findings from the Systematic Treatment Enhancement Program for Bipolar Disorder (STEP-BD study) indicate that 44% of individuals with bipolar disorder who undergo antidepressant treatment experience at least one instance of a hypomanic or manic affective switch. It occurred more likely in patients receiving tricyclic antidepressants and multiple antidepressant trials (40). The SUSTAIN 2 study investigating the long-term safety of ESK-NS in patients with TRD reported no manic switches among adverse events (41). Similarly, the recent study by Martinotti et al. (14) found no differences in affective switches between the TRD and B-TRD groups and only one case in the B-TRD group. In the study by Diazgnanados et al. (15), one patient treated with ketamine and one in the placebo group developed manic symptoms. In the study by Fancy et al. (23) which was a real-world trial three out of 66 patients with bipolar depression presented hypomania after 3 or 4 ketamine infusions, all of them were receiving antidepressants as coexisting treatment. No other cases of the manic/hypomanic switch were reported. Detailed description of all studies discussed above except case reports are included in Table 1.

**Table 1:** Studies investigating short-term add-on ketamine use in bipolar depression.

Ketamine		Study duration and number of patients	Safety	Tolerability	Effect
Racemic intravenous single dose	Diazgranados et al. (14)	14 days *n* = 17 TRBD	One participant in each group developed mania	Treatment was well tolerated. Dissociative symptoms at 40 min point	Significant improvement within 40 min and remained significant through day 3. Response rate 71% at some point during the trial
	Zarate et al. (15)	14 days *n* = 15 TRBD	No serious adverse events (SAE)	Dissociative symptoms, feeling drowsy, cognitive impairment, anxiety, nausea, dizziness, blurred vision, headache	Significant improvement of depressive symptoms and suicidal ideation was observed within 40 min, through day 3. Response rate 79% at some point in the study
	Permoda-Osip et al. (17)	14 days *n* = 18 BD	Not reported	Not reported	Eight patients (44%) responded, on the 7th day after infusion. Improved cognitive performance on day 3
	Grunebaum et al. (16)	6 weeks *n* = 16 BD	Six SAEs occurred, involving suicidal thoughts during FU months 2–6, leading to admission (4:3 for one subject)	Mild dissociative symptoms, mild increase in blood pressure	More than half of patients (57%) demonstrated a response in suicidal ideation. Depression ratings decreased but not significantly
	Rybakowski et al. (18)	14 days *n* = 53 TRBD	No significant adverse events were reported	Transient increases in blood pressure, depersonalization, derealization, in 1/3 of patients only during the infusion	The response rate was 24.5% at 24 h post-infusion, and 51% to at day 7
Racemic Intravenous Multiple doses	Chen et al. 2019 Phase 1 (24)	2 infusions day 1 and day 4, (phase 1) *n* = 30 TRD *n* = 19 TRBD	Not reported	Not reported for the phase 1	Response rate was slightly higher in the bipolar group and reached 68%, whereas in the MDD group it was 63% at day 7 compared to baseline
	Zhuo et al. (23)	9 infusions over 3 weeks *n* = 38 TRBD	Not reported	Not reported	Significant improvement in depressive symptoms after 1 treatment. Relapses occurred during the second week, by 21 days after starting treatment, the patients reported worsening of depressive symptoms compared to baseline
	Zheng et al. (20)	6 infusions over 12 days, 2 weeks FU *n* = 19 TRBD	No SAE reported	There were no significant dissociative and psychotomimetic symptoms	After the first infusion, the rates of response and remission were 21.1% and 15.8%. After the 6th infusion they were 73.7% and 63.2%, respectively
	Wilkowska et al. (21)	8 infusions over 4 weeks, 1 week FU *n* = 13 TRBD	No SAE were observed	Treatment was well tolerated	Following the seventh infusion, 61.5% of participants responded
	Abbar et al. (34)	2 infusions baseline and 24 h *n* = 26 BD *n* = 56 MDD	Eight patients in the placebo and 6 patients in the ketamine arm attempted suicide. In bipolar group: 2 in placebo v 0 in ketamine group	Side effects were limited, no manic or psychotic symptoms, all side effects were rated as minor, and reduced significantly between during first 4 days	Remission rate of suicidal ideation in bipolar group at day 3 was 84.6% compared to 28% in the placebo group. Moreover, a mediating effect of mental pain was found
	Fancy et al. (22)	4 doses 0.50–0.75 mg/k across 8–14 days, 1 week FU *n* = 66 TRBD	No SAE reported	Infusions were well tolerated; hypomania was observed in 3 patients (4.5%). No cases of mania or psychosis	Response rate was 35% and remission rate was 20% after four infusions
Esketamine nasal spray	Martinotti et al. (13)	Two doses per week in the first month, and 1 dose/week in following 2 months 28–84 mg 1 month FU *n* = 35 TRBD *n* = 35 TRD	No SAE reported, no increase in suicidality, no need for hospitalization	Treatment well tolerated. One case of manic switch in TRBD group, 1 case of reported psychomotor agitation in the TRBD causing discontinuation after 2 weeks. Adverse effects were reduced after the treatment without sequelae	In TRBD group, nine subjects (25.7%) responded. in first month (T1) and 24 subjects (68.57%) in third month, (T2) while six patients (17.14%) were remitters in T1 and 17 (48.57%) in T2. No significant differences in response. or remission rates between subjects with TRBD and TRD. Esketamine showed a greater anxiolytic action in subjects with TRBD than in those with TRD
Esketamine subcutaneous	Surjan et al. (31)	One injection per week for 6 weeks 0.5–1 mg/kg *n* = 39 MDD *n* = 31 BD	No SAE reported	Good tolerability profile, adverse events were mild and transient	Significant anti-suicidality effects were observed in both major depressive disorder and bipolar groups, with a rapid onset of action
Arketamine intravenous	Bandeira et al. (32)	Two infusions, 0.5–1 mg/kg mg/kg *n* = 6 BD	No SAE reported	All individuals tolerated both doses, exhibiting nearly absent dissociation, and no manic symptoms	The mean baseline (MADRS) total score was 36.6, which decreased to 27.8 24 h after the first infusion 0.5 mg/kg. In respect of the 1 mg/kg dose, the mean MADRS total score before the second infusion was 32.0, which dropped to 17.6 after 24 h

SAE, serious adverse event; MDD, major depressive disorder; TRD, treatment resistant depression; TRBD, treatment resistant bipolar depression; BD, bipolar depression; MADRS, Montgomery–Åsberg Depression Rating Scale; FU, follow-up; *n*, number of patients included in the study.

## 5 Discussion

This narrative review synthesizes the latest research on the application of ketamine and its enantiomers in bipolar depression, providing evidence in support of their significant antidepressant efficacy. The majority of the studies discussed are open-label involving small cohorts, underscoring the need for more comprehensive research through large-scale RCTs with extended follow-up durations. Presented research also suggest ketamine’s antisuicidal properties with only one study reporting worsening in this symptomatic dimension (17, 23, 32, 35). The risk of the affective switch seems low, and transient self-limiting adverse effects like drowsiness, dizziness, blurred vision, nausea, dissociative symptoms, and headaches confirm the good safety profile of ketamine in BD (12). There is evidence for ketamine rapidly reducing often resistant symptoms like anxiety, irritability, and agitation which consist mixed features domain in TRBD (42). Anhedonia, a profoundly incapacitating aspect of bipolar depression, is associated with heightened risks of suicide, diminished life quality, social isolation, and suboptimal responses to treatment. Research indicates that ketamine possesses properties that counteract anhedonia, which is especially pertinent considering the absence of officially sanctioned treatments for this condition (43, 44). Available studies on MDD patients show more pronounced and sustained antidepressant effects with multiple versus single doses of ketamine (45). The above data suggest it is also true for bipolar depression (14, 21, 23).

The antidepressant effect of ketamine, an NMDA receptor antagonist is achieved through three hypothetical pathways. One engages in the disinhibition of glutamate through an NMDA blockade in inhibitory neurons. A glutamate surge activates α-amino-3-hydroxy-5-methyl-4-isoxazole propionic acid receptors (AMPA) and causes the release of BDNF and mTOR (mechanistic target of rapamycin) which increases the number and function of synapses *in vitro* (46). The second mechanism involves intracellular inhibition of eukaryotic elongation factor 2 kinase (eEF2K), the dephosphorylation of eukaryotic elongation factor 2 (eEF2), and an increase in BDNF translation which allows rapid production of this neurotrophin in the hippocampus (47). The third hypothesis suggests the direct binding of ketamine to the TrkB receptor. All these processes lead to the unique impact of ketamine on neuroplasticity (48). Proposed mechanisms responsible for the ketamine’s antidepressant effect may involve the opioid system and anti-inflammatory response through the kynurenine pathway and cytokine inhibition (49, 50). Repeated dosing of ketamine is hypothesized to activate dopaminergic and noradrenergic neurons, potentially sustaining its antidepressant action (51). Moreover, postmortem examinations and magnetic resonance spectroscopy (MRS) have identified increased levels of glutamate/glutamine (Glx) and lactate in the cingulate gyrus across various affective states in bipolar disorder (BD) patients, including manic, mixed, and depressive states. This elevation is particularly marked in patients with bipolar disorder who exhibit melancholic depression and rapid cycling. The underlying cause is believed to be mitochondrial changes, with the concurrent upsurge in Glx and lactate attributed to disruptions in cerebral energy metabolism and a transition from oxidative phosphorylation to glycolysis (52, 53). Certain researchers propose that dysregulation of the glutamatergic system may represent a characteristic trait of bipolar depression (14). There is evidence for a potential role of glutamatergic transmission compounds in relieving anxiety, anhedonia, and mixed features characteristic of bipolar depression (54). Preclinical evidence suggests that glutamatergic agents can have mood stabilizing properties (55). Hypothetically ketamine and esketamine could stabilize cellular membranes through the modulation of the tonic membrane influx of Ca^2+^ and Na^+^ (56). Preclinical evidence shows that esketamine and arketamine can inhibit voltage-gated sodium channels (VGSC), which is a property specific for mood stabilizers like valproate and lamotrigine (57). Moreover, ketamine modulates the glycogen synthase kinase 3β (GSK-3β) pathways, which is hyperactive in unipolar and bipolar subjects. Interestingly this mechanism is common with lithium (58). It is also possible that ketamine augments the effect of lamotrigine although this hypothesis needs further studies (59).

**Figure 1:**
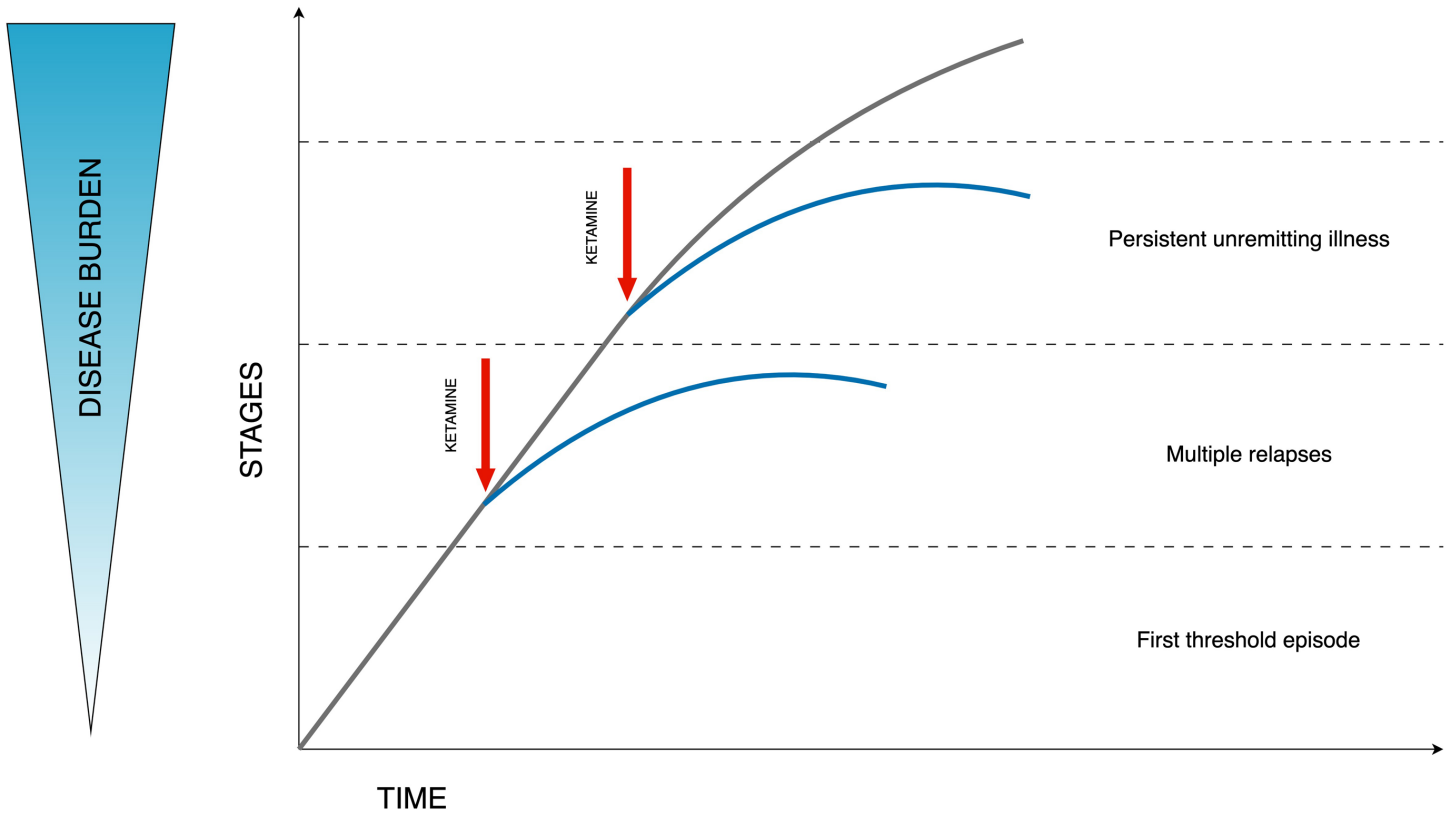
Hypothesized effect of ketamine on the disease burden in bipolar depression. Stages described according to Berk staging model stages: 3b, 3c, and 4 (60). Figure adapted from Wilkowska and Cubała (64).

The progressive character of bipolar disorder is captured in staging models (60). Based on the consensus from the International Society for Bipolar Disorders (ISBD) Staging Task Force the application of clinical staging in psychiatry is currently theoretical. Despite its common usage in general medicine, the application in psychiatry is hindered by a significant gap in understanding the origins, and disease mechanisms, and a lack of concrete structural or biological indicators. Nonetheless, there is optimism that clinical staging might enhance the timely detection and diagnosis and improve decision-making regarding treatment strategies. Additionally, it has the potential to streamline research discussions concerning the clinical and pathological aspects of this diverse array of disorders. The necessity for more extensive research in this domain is clear (61). The principal evidence supporting the biological underpinnings of clinical staging in psychiatric disorders points to persistent inflammation and neuroanatomical alterations that may precipitate cognitive decline. Additional biological mechanisms, including modifications in telomere length indicative of DNA changes and various cellular activities, are also implicated in this context (62, 63). Evidence suggests that ketamine administration may result in enhanced dendritic spine formation, augmented hippocampal volume, modulation of functional connectivity, and effects on inflammatory and glial modulation. Consequently, the concept of “downstaging” in TRD has been proposed, which could be applicable to bipolar disorder as well, given its progressive nature (64). This concept suggests the possibility of formal disease stage reduction through the transformative effect of ketamine treatment increasing the functional reserve and diminishing the degree of treatment resistance. The above hypothesis is illustrated in Figure 1.

The extant data suggest that ketamine, along with its enantiomers, may have the potential to substantially alleviate the significant disease burden associated with bipolar depression (2).

## 6 Conclusion

This review provides support for short-term ketamine use in bipolar depression. However, the literature is limited and must be taken with caution. Data on esketamine use in MDD constituting psychiatric emergency indicate favorable safety and tolerability profile. With several unmet needs in bipolar depression regarding the efficacy and treatment effect onset, short-term ketamine use in bipolar depression as an add-on treatment may be a promising option for severity abatement, and social incapacity duration. Considering available data and an urgent need for more effective treatments we call for more research on this group of patients to enable approval for short-term ketamine treatment in bipolar depression.

## Author contributions

AW: Data curation, Investigation, Visualization, Writing – original draft, Writing – review & editing. WC: Conceptualization, Funding acquisition, Investigation, Methodology, Supervision, Writing – review & editing.
